# Hungarian Managed Care initiatives between 2000 and 2007: regional health outcomes of the Hungarian Care Organizations

**DOI:** 10.1017/S1463423618000701

**Published:** 2018-10-08

**Authors:** Csaba Móczár, Imre Rurik

**Affiliations:** 1 Irinyi Health Center, General Practice, Kecskemét, Hungary; 2 Department of Family and Occupational, Medicine, University of Debrecen, Debrecen, Hungary

**Keywords:** care managing, health care reform, health insurance, managed care, risk selection

## Abstract

**Background:**

In Hungary, since 1990, each government has tried to transform and rationalize the structure of health care. One of the reforms was the Care Managing Organization (CMO) programme introduced in 1999.

**Objectives:**

The aim of this paper is to describe the regional, environmental, structural and preliminary health related outcomes of the CMO in Bács-Kiskun County (Central-Eastern Hungary).

**Methods:**

First, cardiovascular screening programmes were organized for pre-screened and randomly selected populations of a total of 4462 persons. Seven years after completing the programmes, regional mortality data were analysed and compared. Second, nutritional and lifestyle counselling programmes with increased physical activity were organized for 2489 overweight or obese patients from the participating primary care practices. Anthropometric and laboratory data were examined after one and two years.

**Results:**

First, for persons with higher cardiovascular risk, appropriate medical treatment was introduced, and after seven years, their mortality rates proved better than the regional and national data. Second, almost all measured anthropometric parameters improved (body mass index, body weight decrease) after the first year and this trend lasted till the end of the second year.

**Conclusions:**

According to the data of the National Health Insurance Fund, the average savings rate for all CMOs for the fiscal years 1999–2007 was 4.94%. The highest rates of savings were realized in chronic and acute inpatient care and medical devices. In the end of 2008, by which time 14 CMOs had already covered 2.1million people, the programme was discontinued by the government, without a comprehensive evaluation of the experience and outcomes.

## Introduction

In Hungary, after the political regime changed in 1990, the health system brought on anomalies characterized by the previous period, such as existing deficiency of resources, and a large-scale waste of sources in addition. Since then, each government has tried to transform and rationalize the structure of health care. One of the reforms was the Care Managing Organization (CMO) programme introduced in 1999 (43/1999. (III.3), 1999).

The declared aims of the CMO programme were

- to monitor and coordinate the health care provision through the entire range of services;
- to emphasize prevention and health education supporting healthy lifestyle;
- to encourage the provision of care in the most appropriate setting and by the most appropriate provider;
- to promote the cost-effective use of services through aligning incentives;
- to strengthen the primary and outpatient care.

The Hungarian health care system is a solidarity-based national health insurance system with mandatory participation for every citizen. There is only one purchaser, the National Health Insurance Fund (NHIF) and employers and employees pay health insurance contribution to this fund that is complemented by general budget transfers (Boncz, 2004).

The central government owns most hospitals (inpatient care) and many of secondary (specialist) providers. Some of them are owned by local municipalities. There are only a limited number of private services. All government-financed health care providers have a service contract with the NHIF, which is a prerequisite for any payment by the government. The NHIF uses different payment methods for providers at different levels of provision. General practitioners are paid mostly on *capitation* with some additional fees. Outpatient care is financed on a *fee-for-service* basis, while inpatient care on a *diagnosis-related group* system (Fetter *et al*., 1980; Boncz *et al*., 2004; 9/1993 (IV.2.), 1993).

This CMO programme was an experimental reform, introducing elements of managed care. The concept was closer to ‘GP’s fund holding’ in the United Kingdom than to *Health Maintenance Organizations* in the United States. In terms of techniques used to control cost and improve efficiency, the US managed care was an experience, the ‘toolbox’ for Hungarian reform in the nineties (Lagoe *et al*., 2005).

The Ministry of Health launched a bid and the CMOs were involved in an application process, thereafter they were systematically selected by the NHIF. Every CMO had a virtual budget, an adjusted capitation account, determined by the number and characteristics of the population they covered. Enrolment was initiated by the family physicians/general practitioners (GPs) and not by the individual patients; therefore, there was no room for risk selection at the patient level. Patients who did not want to be enrolled into the CMO had an option to change their GPs to another one not involved in the programme (Nagy and Tóth, 2006).

The CMO took responsibility to arrange the whole spectrum of health services for the local or sub-regional population having enrolled to the participating GPs. However, patients were still free to choose specialists and hospitals including those that were not contracted with the CMO. The actual service providers were paid by the NHIF according to the national payment system, and then charged the virtual budget of the CMO for all paid services of the population covered by the respective CMO. The idea was to provide care at the least expensive level that is appropriate for the patient’s condition. Typically, the CMOs ran an integrated information system to monitor all patient-related clinical and financial data and to analyse performance against benchmarks (Faragó and Járvás, 2006).

Based on a risk-adjusted capitation from the NHIF, the revenues of the CMOs covered primary, outpatient and inpatient care including imaging (CT, MRI), renal dialyses, dental services, home care, drug subsidies for outpatient care, balneology and transportation of patients (Nagy *et al*., 2006). The capitation formula was weighed by age and sex; therefore, it had only limited predictive power for actual costs. The only exception was renal dialysis, for which an additional payment was added to balance the financial risk. It should be emphasized that risk-adjusted capitation was just a theoretical revenue in a virtual budget of the CMOs, and not an actual payment. Separately from the virtual capitation rate, there were two payments made directly to the bank account of the CMOs – a fee covering the additional costs of the management employed by the programme, and a fee covering the extra costs of mandatory prevention programmmes, activities in the screening that the organizers were obliged to provide. The expenditures of the CMOs included all health care-related payments of the patients that occurred at any level of the health system.

At the end of the fiscal year, the NHIF calculated the financial balance of the CMOs by comparing enrolees’ per capita rates to use of services. If the financial balance resulted a surplus, part of the savings was transferred to the CMOs as a cash payment. For CMOs with deficit, there were no penalties or other fiscal consequences. CMOs were allowed to use savings to make incentive payments to their contracted GPs and staff members. Since 2003, CMOs were obliged to set up a complex reward system including incentive payment not only for their GPs and staff members but also for the contracted partner providers of the CMOs that played significant roles in the medical care of the enrolled patients (Kovácsy, 2003). The average savings rate for all CMOs for the fiscal years 1999–2007 was 4.94%. The highest rates of savings were realized in chronic and acute inpatient care and medical devices. The saving rate of CMOs in Bács-Kiskun County was around 0.12% during the programme (Falusi *et al*., 2006).

The CMO programme ran until 2008, thereafter the government stopped it without a systematic analysis of its findings and health-related outcomes.

Previous publications describing the programme were unable to perform these tasks, without available local data ready to be compared to nation-wide data (Boncz *et al*., 2003; Kerekes, 2003; Sinkó, 2004; Dankó *et al*., 2005; Donkáné Verebes and Oberfrank, 2005; Belicza *et al*., 2006).

## Aim

The main scope of this paper is to describe the outcomes of a participating CMO in Bács-Kiskun County (Central-Eastern Hungary), where local data were available and other data were collected from the national database. Methods, data and results of (I) cardiovascular screening and (II) lifestyle programme organized for overweight and obese persons are described and analysed in this paper.

## Methods

### Cardiovascular screening programme

#### Selection of participants

The programme, focussing on screening and prevention of cardiovascular diseases, was started in Bács-Kiskun County in 40 primary care practices.

#### Inclusion criteria

Patients without any known cardiovascular disease and without any medication were eligible.

##### Pre-screened group

Patients were selected from a group who had not been seen by their GP for more than two years and whose medical history contained at least one of the followings:

- male over 55, female over 65 years of age;
- cardiovascular event (acute myocardial infarction or stroke) in the family medical history;
- smoker;
- body mass index (BMI) above 25 kg/m^2^ (measured during previous encounters).

##### Control group

Family doctors were authorized to involve randomly selected patients without any known cardiovascular disease and who did not meet the other specific criteria mentioned above.

#### Exclusion criteria

Exclusion criteria were pregnancy and age below 18 years. Patients who met the inclusion criteria were invited by post.

#### Screening procedures

A data sheet was filled in with the following data:

- personal medical history coded according to ICD-10 (International Classification of Diseases, 2017);
- cardiovascular diseases and/or diabetes mellitus in the family history;
- questions about lifestyle: smoking, alcohol consumption, physical activity;
- anthropometric measurements (waist circumference, body height and weight, calculation of BMI);
- laboratory tests (fasting blood glucose, triglyceride, total cholesterol, HDL and LDL- cholesterols, C-reactive protein).

Special questions were focussed on the eating habits of patients, with meals, applicable for Hungarian circumstances.

#### Ethics

Since the aim of the programme was not scientific and was based on governmental decree, ethical approval was not required. Patients were asked to sign the Informed Consent Form, approved by the Regional (Szeged) Ethical Committee of the Hungarian Medical Research Council (30 June 2003).

#### Intervention

The intervention depended on the cardiovascular risk status of patients. When a cardiovascular disease or diabetes was diagnosed, the appropriate medical treatment started, based on national guidelines (Hungarian Diabetes Society, 2004; Hungarian Society of Hypertension, 2005).

Patients with high risk were initiated to receive a tailored follow-up therapy and received personalized information about a healthy life style (First Hungarian Cardiovascular Consensus Conference, 2003).

#### Mortality data

After finishing the programme in 2006, the benefits of screening for the local population were estimated by the comparison of the mortality data of CMOs (participating family practices) and the regional and national mortality trends based on the data of the National Census of 2011 (Census 2011-Regional data-3.2 Bács-Kiskun County, 2011).

##### IT support available for the project

Appropriate software was developed for GPs, based on practice data, providing access to laboratory data and individualized description of recommended life style changes, estimated by SCORE points (De Backer *et al*., 2003).

#### Statistics

Student’s *t*-test was used to compare groups. Besides the crude mortality ratio (death/1000 people), standardized mortality ratios were calculated with regards to the number of registered deaths compared to the expected number of deaths in the given population based on Hungarian age- and gender-specific mortality data of 2011 (Census 2011-Regional data-3.2 Bács-Kiskun County, 2011).

### Lifestyle programme for overweight and obese people

#### Selection of patients

From the 29 primary care practices of CMOs 2489 patients were recruited.

#### Inclusion criteria

Overweighed and obese persons above 18 years old were selected and invited by their own GPs.

#### Measurement and history taking

Anthropometric data were precisely measured, and BMI was calculated. A data sheet was filled including the age, gender, past medical history, type of occupation, living in or without (self-rated) stressful circumstances, amount and type of regular physical activity, smoking habits, alcohol consumption, previous and recent medication. It was followed by laboratory measurements.

Special questions were focussed on the nutritional habits of patients, applicable for Hungarian circumstances (Jancsó *et al*., 2011). A nutritional diary was kept by every patient including two workdays and one weekend day, combined with an eating frequency questionnaire. These data were analysed and considered as basis of an individual diet. The energy needs of patients were calculated individually, based on body weight, metabolic rate, and physical activity.

#### Intervention

Patients participating in the study were managed by a team, including their own GP, a nurse, a dietician and a physiotherapist. If needed, this team worked out and offered an individual plan for lifestyle changes and nutritional recommendations, based upon a guideline (Pados, 2001).

Two ways of intervention were offered for the participants; diet and lifestyle changes with increased physical activity. Diet was counselled by dieticians and/or experienced nurses. They provided special recipes for cooking and preparing dishes, helped to estimate the real energy needs, and planned personalized low energy menus to develop a healthy and balanced individual diet. Specific handouts and booklets were distributed as well. Family doctors, health and sport educators gave advice on how to increase physical activity. Principles of the recommended exercises were drilled with special attention to the gradually increasing intensity. Patients were advised to perform dynamic exercises, at least 30 min/day (walking, jogging, running, cycling, and gymnastics). The exercises started from 50 to 60% of the submaximal pulse rate and was increased step by step every three weeks to attain 80–100% of the submaximal heart rate. A slower increase of physical activity was planned, if a patient’s general condition or cardiovascular status made it necessary. With patents who were already involved in a medication cure, the treatment and the lipid lowering therapy were not considerably modified. All the medications were constantly controlled by patients’ own GPs. The consultations were personalized (Apor, 2001). This activity was helped by existing local patients’ clubs, or temporary clubs were organized, supported by local authorities.

##### Controls and follow-up

Anthropometric parameters, pulse rate, blood pressure in rest and after exercise were usually checked after the third and sixth months, and were repeated at the end of the first and second years, when laboratory parameters were also measured.

#### Statistics

We interpolated BMI-time lines into each patient diagrams one by one using linear regression, thus reducing the row of BMI to two items of data: baseline BMI and the rate of change in BMI. Later these two items of data were compared to other parameters by two methods: decision tree and covariant analysis. Changes of BMI were analysed by a Wilcoxon signed-rank test.

## Results

### The results of the cardiovascular screening programme

#### The characteristics of the population and the main differences between the groups

Altogether 4462 patients were screened (3420 in the pre-screened group) 1977 (1518) men and 2485 (1902) women. The average age was 47.4 years (47.9 for men and 47.1 for women), median: 49 years. Participants within the pre-screened group were older (mean:48.9±9.8 versus 40.9±9.6), had significantly higher systolic blood pressure, total and LDL cholesterol values, fasting plasma sugar levels, body weight, and SCORE values in both genders, higher BMI and waist circumferences in men. They also had higher hs-CRP and triglyceride values, without significant statistical differences.

The average scores were significantly higher in the case of the pre-screening approach. Higher numbers of high-risk patients were found in the pre-screened group, compared to the randomly screened population (17.4 versus 0.6%).

#### Retrospective aggregate mortality data

Aggregated mortality data were collected from the practices after the programme had been completed. The average length of follow-up was 7.15 years. Data contain all causes of mortality and cardiovascular mortality, separately. We had information about 4182 of the 4461 screened patients. The total number of deaths was 158, which is a mortality of 5.7%° (national: 12.0%_o_), cardiovascular death rate was 46 patients, which is a mortality of 1.3%° (national: 6.4%o). Table 1 shows the summarized mortality data of participating practices.

**Table 1:** Comparison of the mortality data of participating practices

	General mortality			Cardiovascular mortality		
	Crude	SMR		Crude	SMR	
Hungarian national	12.0	100.0		6.9	100.0	
CMO practices	5.7	32.0	*P*<0.001	1.3	28.8	*P*<0.001

SMR=Standardized Mortality Ratio; CMO=Care Managing Organization. SMR was calculated based on Hungarian sex- and age-specific mortality rates in 2011 (Census 2011-Regional data-3.2 Bács-Kiskun County, 2011). Crude: death/1000 inhabitants; SMR: %.

The averages of standardized mortality ratios of cardiovascular diseases were 0.3817 in men and 0.4409 in women during the programme (between 1999 and 2008), and they increased to 0.42 and 0.5505 thereafter (2009–2012). This change was significant among women (*P*=0.0029, SD: 0.04).

### Lifestyle programme for overweight and obese people

From the CMOs 2489 patients of 29 primary care practices were recruited.

During the follow-up periods of one year 1793 (72%) were under control and 901 (36%) completed the programme after two years.

#### Anthropometry

At the baseline, BMI was higher in patients with diabetes mellitus, hypertension, age over 40 years, when higher sugar intake, more fat-rich meals, less fruit, and unhealthy diet were reported in nutrition-questionnaires. Those who did not undertaken previous physical activity, did not smoke, reported stressful life and were residents in cities or larger villages. The average recorded daily energy intake of these patients was 24% higher than their estimated requirements.

While the only inclusion criteria were obesity and overweight, higher energy intake gave a feasible explanation for these conditions.

##### BMI

The BMI decreased continuously and significantly in the first year, as seen in Table 2.

**Table 2:** Change of body mass index (BMI) and waist circumference

	Men					Women				
		First year change		Second year change			First year change		Second year change	
	Baseline	Mean (SD)	*P*	Mean (SD)	*P*	Baseline	Mean (SD)	*P*	Mean (SD)	*P*
BMI (kg/m^2^)	32.81	−0.3 (3.0)8	<0.001	−0.009 (3.8)	ns	31.98	−0.66 (4.2)	*P*<0.001	−0.008 (3.7)	ns
Waist circumference (cm)	106.35	0.0 (14.0)	ns	(16.5)	ns	98.42	0.0 (14.2	ns	−0.09 (16.5)	ns

The initial body weight and BMI decreased after the first year, mostly with women. These changes were positively accepted by the patients.

##### Waist circumference

There were smaller and not significant changes in this parameter. It was higher in the case of persons with higher initial data, hypertension, or/and diabetes mellitus, in males, in patients older than 40 years, in those with a salt intake, and those who did not perform regular physical activities and lived stressfully in larger cities or villages. In patients whose anthropometric parameters were measured after the second year, similar data were registered without significant differences.

##### Haemodynamic parameters

The blood pressure of the participants decreased following the intervention, significantly in men, after the first year (Table 3.).

**Table 3:** Changes of resting systolic and diastolic blood pressure

	Men					Women				
		First year change		Second year change			First year chenge		Second year change	
	Baseline	Mean (SD)	*P*	Mean (SD)	*P*	Baseline	Mean (SD)	*P*	Mean (SD)	*P*
Resting systolic blood pressure (Hgmm)	140.6	−6.9 (36.0)	<0.001	−0.11 (36.5)	ns	135.8	−4.8 (32.0)	*P*<0.001	−0.22 (33.5)	ns
Resting diastolic blood pressure (Hgmm)	83.0	−0.001 (10.0)	ns	−0.008 (8.0)	ns	81.0	0.01 (9.8)	ns	−0.006 (8.0)	ns

#### Metabolic parameters

##### Fasting blood glucose

It decreased significantly by the end of the first year. The initial levels of blood glucose were higher in older persons, in patients with diabetes, hypertension, and higher alcohol and salt consumption. These values decreased more intensively in patients with higher initial levels and/or higher triglyceride, and who were under 45 years of age, preferred fatty meals and lived stressfully.

##### Total cholesterol

During the follow-up period, total cholesterol significantly decreased in the majority of patients in both consecutive years. The decrease was higher in patients with higher initial levels and who were younger than 45 years.

##### HDL-cholesterol

The level of HDL-cholesterol decreased in most of the patients, similarly to other cholesterol fractions. The initial level of HDL-cholesterol was higher in females, who were less obese, did regular physical activities, lived in a big city, suffered from psychiatric diseases, did not eat fruits regularly and had higher initial level of triglyceride.

##### Triglyceride

By the end of the first year, the triglyceride level decreased significantly in half of the patients. The improvement was more visible under of age of 45, in persons with higher initial levels, in those who did regular physical activity, had meals four to five times a day, preferred fatty meals and lived in less stressful circumstances.

A strong correlation was experienced between metabolic parameters. The worse the initial conditions of the patients were, the higher improvement was registered during the programme. Positive changes often went parallel and patients had more benefits and expectable improvement in health, and a decrease in cardiovascular risk. With 36% of the patients who completed the programme, the average of registered laboratory parameters was similar to that of the first year, while wide interpersonal differences were observed.

From 5 to10% of weight loss within a few months usually leads to a significant reduction of cardiovascular risk. The small differences between cities and villages could be explained with higher physical activity of the rural people.

## Discussion

There was little doubt that US managed health care initiatives have been followed with considerable interest in Europe. It was not clear, however, if the current US models are or can be applicable without major modifications. In Europe universal and mandatory health care is a basic principle, and health care systems remain a national responsibility even within the European Union. Reforms in individual countries have remained partial and uncoordinated, and priorities for change differ. The classic CMO model focuses on GPs, and well-functioning GPs can produce a greater saving in the system. In several countries (United Kingdom, the Netherlands), the gatekeeper role of GPs has been strengthened, or in Baltic countries, GPs were strengthened, and hospital-centred health care was eased (Erdmann and Wilson, 2001; Leukert-Becker and Zwiefel, 2014).

The programme of establishing CMOs was a comprehensive model programme focussing on the Hungarian health care system as a whole. In 2005 the number of enrolees were more than 2.2 million, 22.5% of the total population of Hungary. CMOs were mostly focussed on savings and tried to form a cost-effective care system, and they emphasized the reduction of unjustified expenses instead of increasing revenues.

This CMO pilot programme generated debates during the running period, some experts considered it as incompatible with the Hungarian solidarity-based health insurance system. Others thought that it could serve as an important way for a comprehensive health reform (Sinkó, 2002; Belicza *et al*., 2004; Jóna *et al*., 2010).

The health outcomes of the programme were only generally formulated. The State Audit Office of Hungary agreed that no health outcome indicators were identified at the start of the programme (State Audit Office of Hungary, 2005). The prevention programmes were obligatory for every CMO, but they only received professional guidance later in 2003 to prepare their programmes to fit into national health programmes.

Up till now, no analysis has been published about the measured outcomes of the programme.

The prevention programmes organized by the CMOs had an unquestionable impact on the health indicators of the enrolled population. The high cardiovascular mortality and obesity has been a serious problem in Hungary since many decades (Rurik *et al*., 2016). On one hand, we could explain that a well-organized, controlled, and sufficiently funded screening programme could be a basis of an effective cardiovascular prevention. Risk score assessments can help to identify patients with high risk, to avoid numerous unnecessary examinations. The ratio of patients with higher risk scores was considerably more frequent among the pre-screened population, so this method proved ways more cost-effective.

Mortality was lower in the participating primary care practices where screening programmes were performed than the average national figures. The prevention programmes focussed mainly on the prevention of cardiovascular diseases and could be considered as effective.

Although a good cooperation was established between CMOs and other health service providers, there were savings in health care expenditures, participating health care professionals were better paid and motivated. Unfortunately, the government ceased the programme without explanation. Politicians did not consider this system as a model for the improvement of the Hungarian primary care and public health services. A development of GPs started (privatization, only a registered general practitioner could establish a praxis, officially recognized general practitioner exam) in the 1990s, and a further step in this process could have been a network of GPs with an important role in the organization of local health care within the framework of CMOs. Since the CMOs were terminated, Hungarian health care policy has not been able to move on to strengthen primary care.
